# Associations between the menstrual cycle, lifestyle factors and clinical assessment of the ocular surface: a prospective observational study

**DOI:** 10.1186/s12905-020-0894-z

**Published:** 2020-02-07

**Authors:** Luisa H. Colorado, Katie Edwards, Lynne Dinh, Sarah Ha, Danica Liu, Annie Luu, Shona Trang, Tina H. Yu-Ting, Katrina L. Schmid

**Affiliations:** Institute of Health and Biomedical Innovation, School of Optometry and Vision Science, Queensland University of Technology, Room Q-504, Level 5, Kelvin Grove, Queensland 4059 Australia

**Keywords:** Dry eye, Menstrual cycle, Lifestyle factors

## Abstract

**Background:**

Little is known about the ocular surface changes over the menstrual cycle in young women and the interactions with lifestyle factors. Therefore, the purpose of this study was to explore the associations between modifiable lifestyle factors and menstrual cycle phases on the ocular signs and symptoms of dry eye in young healthy women.

**Methods:**

This was a prospective 1-month observational study. Thirty young healthy women with regular, 24 to 32-day menstrual cycles were recruited. Participants attended three visits at day 7, 14, and 21 (± 1) of their menstrual cycle. At baseline, general health questionnaire was conducted. At each visit, symptomology was quantified using Ocular Surface Disease Index (OSDI) and overall ocular comfort (OOC, visual analogue scale). Ocular signs were assessed using Efron scales, tear break-up time (TBUT) and phenol red thread (PRT). Pearson’s correlation was used to determine associations between variables at each visit.

**Results:**

A total of 26 participants (mean age = 22.3 ± 3.7 years) with an average menstrual cycle of 28.3 ± 1.3 days completed the 3 visits. The interaction between signs/symptoms and lifestyle factors changed over the cycle. At the follicular phase (day 7), lifestyle factors such diet and levels of stress were correlated with PRT and OSDI, (r = − 0.4, *p* = 0.022; r = 0.4, *p* = 0.045 respectively). At the ovulation phase (day 14), the general health score was correlated with OOC scores (r = 0.4, *p* = 0.047). At day 14, exercise frequency correlated with PRT (r = − 0.4, *p* = 0.028) and caffeine intake was positively correlate with both; TBUT (r = 0.5, *p* = 0.020) and PRT (r = 0.5, *p* = 0.014). At the luteal phase (day 21), we found no correlations between lifestyle factors and dry eye signs or symptoms.

**Conclusions:**

The associations between lifestyle factors and objective and subjective ocular surface assessment appeared to be more pronounced during the ovulation phase of the menstrual cycle compared to the follicular and luteal phases. Misalignment of these factors with the ocular health during the luteal phase could be attributed to central sensitization and changes in levels of luteinising hormone. Natural hormonal changes during menstrual cycle should be considered for diagnosis and treatment of dry eye in young healthy women.

## Background

Dry eye occurs when the quantity and/or quality of tears fails to keep the surface of the eye adequately lubricated. The approximately 30% global prevalence of dry eye has increased in recent years and the diagnosis and treatment for this multifactorial disease is still a challenge [1]. A reason for this includes the poor correlation between ocular signs and symptoms [2]. Risk factors have been categorized as modifiable/non-modifiable by the Tear Film and Ocular Surface Society (TFOS) and Dry Eye Workshop (DEWSII) [3]. Age and gender are non-modifiable risk factors for dry eye diseases. Evidence of this suggests that females, particularly over 40 years, are greatly impacted by the diseases in comparison to males (of the same age) in part because of the hormonal changes throughout life. However, little evidence on clinical sign and symptoms of dry eye exist in young healthy females. Although hormone intake is considered as a modifiable risk factor for dry eye, the effect of natural hormonal changes in levels of estrogen and progesterone during the regular menstrual cycle in dry eye is little understood, especially in young adults.

Oestrogen and progesterone are the key hormones involved in menstruation and levels rise and fall twice during the cycle. While the estrogen is responsible for growing and maturing the uterine lining and the egg before ovulation, the progesterone, also called the relaxing hormone, balance the effects of estrogen by controlling the build-up of the uterine lining. Oestrogen is most abundant in the first half of the menstrual cycle and progesterone dominates the second half of the cycle. Thus, the menstrual cycle has three key phases, after menstruation, linked to hormonal changes.

During the follicular phase (day~7) the hypothalamus stimulates the pituitary gland to release a hormone called follicle stimulating hormone (FSH). This causes a follicle within one of the ovaries, containing an egg, to mature. As this follicle matures, an increase in oestrogen is initiated. In the ovulation phase (day~14) the cycle occurs as oestrogen peaks. This causes the pituitary gland to release a surge of luteinising hormone (LH). This hormone causes the ovary to release the matured egg which is swept into the fallopian tube. The luteal phase (day~21) occurs immediately after ovulation and continues until the end of the cycle. During this time the released egg travels towards the uterus to become fertilized. Progesterone levels also rise during this phase in order to prepare the womb in anticipation of the egg becoming fertilized. However, if the egg does not become fertilized, the levels of oestrogen and progesterone begin to decrease rapidly. The drop in these hormones initiates contractions of the smooth lining, and the menstrual phase begins again.

Induced effects of oral contraceptives have been reported in sensory systems such as olfactory epithelial cell maturation, indicating that premenopausal women who take 30 μg of ethinyl etradiol, presented an increased respiratory epithelial cell maturation index compared to those taken only half of the doses [4]. Another sensory organ altered by the effect of contraceptives is the olfactory, when measuring sensitivity thresholds using a variety of odours [5]. Auditory brainstem response also seems to be affected as shown in a group of women taken the pill compared to the control group (no taken the pill) over the menstrual cycle [6].

In the human eye, estrogen and progesterone receptors are present in the ocular surface including the tears [7]. The level of these hormones plays a role in tear production and blinking rate (number of blinks per minute) as demonstrated in females taking birth control pill who showed less tear production and higher blinking rate compared to an age-matched control group under no effects of the pill [8]. Subjective dry eye symptoms, assessed using the questionnaire for Ocular Surface Disease Index (OSDI), and ocular signs, such as corneal sensitivity, tear volume and tear evaporation time measurements (tear break-up time test) worsen during the follicular phase of the menstrual cycle [7, 9, 10]. These changes across the menstrual cycle happen naturally and could be considered as non-modifiable risk factors for dry eye.

Other “modifiable” risk factors contributing to dry eye include sedentary lifestyles, environmental conditions, smoking, poor nutrition, caffeine intake and work demands [1, 3, 11, 12]. Additionally, the extended hours spent performing visual tasks such as computer work, television, reading and the use of tablets/mobiles and other devices can also aggravate sign and symptoms [13]. It is suggested that during these visual tasks, a diminished blink frequency rate and incomplete blinking contribute to accelerated tear evaporation, leading to dry eye [3].

The associations between modifiable and non-modifiable risk factors for dry eye diseases during the menstrual cycle have never been explored before in young healthy adults. Therefore, this study sought to determine if the phase of the menstrual cycle (a non-modifiable risk factor) altered the clinical assessment of the ocular surface and the observed variations associated with modifiable (e.g. lifestyle) risk factors. This information could contribute to determine better diagnosis of dry eye diseases in young females.

## Methods

### Study design and participants

Participants were recruited from the staff and student population of the Queensland University of Technology, Brisbane, Australia. Ethical clearance was provided by the Queensland University of Technology Research Ethics Committee and written informed consent was obtained from all participants before enrolment. The research was conducted in accordance with the principles of the Declaration of Helsinki.

This was a prospective 1-month observational study. Thirty young healthy women with regular, 24 to 32-day menstrual cycles were recruited. Participants attended three visits at day 7, 14, and 21 (± 1) of their menstrual cycle. A 3-day window (the calculated date and 1 day either side) was used to accommodate for subject availability and decrease dropout rates. A forward counting method from the first day of the last menstrual cycle was implemented to calculate day 7, 14 and 21 days of the next cycle [14].

At the screening visit, the general health questionnaire was conducted, and inclusion/exclusion criteria was applied. Individuals were not eligible if they had active ocular surface disease, treatment for any systemic condition that may affect the ocular surface, used of anti-inflammatory medication or were pregnant or lactating.

All three visits involved reporting the frequency of lifestyle choices made during the week prior to the visit. Ocular symptoms were assessed using OSDI and overall ocular comfort (OOC, using a 0–100 visual analog scale). Ocular signs were measured using ocular surface assessment (Efron grading), and tear quality and quantity was assessed using tear break-up time test (TBUT) and phenol red thread (PRT) respectively.

Multiple associations between modifiable and non-modifiable lifestyle risk factors and ocular health during the menstrual cycle have never been explored before in young healthy adults, and for this reason this study is considered as an experimental pilot study. Previous statistical studies have suggested that 10 to 30 individuals constitute a reasonable sample size for a pilot study [15, 16] and therefore 30 healthy participants were recruited and correlations were considered statistically significant for *P* values < 0.05.

### Lifestyle assessment

The lifestyle frequency scores were obtained by gathering information on the personal lifestyle choices made (over the week prior to the visit) related to nutritional options, caffeine intake, exercise frequency, stress levels, hours of sleep, environmental exposure time (outdoors) and hours of device use. A 5-point Likert scale was used and the sum of all questions related to each variable was considered as the total section score. All questions were taken from validated questionnaires [17–23]. There were 2 to 8 questions per variable, with a lower score always representing the healthier lifestyle choice (Table 1).

**Table 1:** Lifestyle factor questionnaire

Nutrition	
• How many times have you chosen a diet low in fat, saturated fat, and cholesterol?	
• How many times have you limited the use of sugars and food containing sugar? (i.e., sweets)	
• How many days have you eaten 2–4 servings of fruit each day?	
• How many days have you eaten 3–5 servings of vegetables each day?	
• How many days have you eaten 2–3 servings of food high in Omega? (i.e. fish, flaxseed, chia seeds, walnuts)	
• How many days have you taken any Omega supplements? (i.e. fish oil capsules)	
• How many days have you drunk more than 1–2 alcoholic drinks? (one drink = 375 ml beer, half glass of wine, one shot of hard liquor)	
Caffeine	
• How many days have you had caffeinated beverages? (i.e. coffee, tea, Coke, energy drinks)	
• How many times in a day have you had caffeinated beverages?	
• How many cups in a day have you had each time? (i.e. 1 cup = 1 shot of coffee)	
Exercise	
• Have you exercised vigorously for 20 or more minutes at least 3 times? (i.e. brisk walking, bicycling, aerobic dancing, using a stair climber)	
• Have you taken part in light to moderate physical activity for 30 or more minutes at least 5 times? (i.e. sustained walking, pilates, hiking)	
• Have you taken part in leisure-time (recreational) physical activities?	
• Have you done stretching exercises at least 3 times?	
Stress	
• How many times have your emotions stopped you from carrying out day-to-day activities?	
• How many times have you felt emotionally drained?	
• How many times have you avoided your study/ work/ commitments and responsibilities?	
• How many times were your hands sweaty (due to stress)?	
• How many times you couldn’t breathe (due to stress)?	
• How many times did you feel lazy when it came to your study/ work/ commitments and responsibilities?	
• How many times have you had trouble concentrating?	
• How many times have you had difficulty eating (due to stress)?	
Sleep	
• How many days have you taken medicine (prescribed or over the counter) to help you sleep?	
• How many times have you had trouble staying awake while driving, eating meals or engaging in social activity?	
• How many times have you woken up (without being physically interrupted) during your sleep?	
• How would you rate your quality of sleep?	
• How many days do you get 8 h of undisturbed sleep?	
Environment	
• How many hours have you spent outdoors each day?	
Device used	
• How many hours per day have you spent watching television?	
• How many hours per day have you spent doing near work activities? (i.e. computer work, using iPad, reading a book, paperwork)	

### Ocular symptoms

#### Ocular surface disease index (OSDI)

The OSDI includes three subscales: ocular discomfort (OSDI-symptoms), which includes symptoms such as gritty or painful eyes; functioning (OSDI-function), which measures limitation in performance of common activities such as reading and working on a computer; and environmental triggers (OSDI-triggers), which measures the impact of environmental triggers, such as wind or drafts, on dry eye symptoms. The questions were asked with reference to a 1 week recall period, and responses refer to the frequency of the disturbance. Responses to the OSDI were scored using the methods described by the authors [24]. Subscale scores were computed for an overall averaged score. OSDI subscale scores can range from 0 to 100, with higher scores indicating more symptoms. The overall OSDI score defined the ocular symptoms as normal (0–12 points) or as having mild dry eye (13–22 points), moderate (23–32 points), or severe (33–100 points) disease [25].

#### Overall ocular comfort (OOC)

An overall ocular comfort (OOC) score was obtained using a 0 to 100-point visual analog scale. The participant was asked a single question, i.e. to rate the overall comfort of their eyes during the week before the visit. Participants were presented with a line that was 100 mm long, with faces and descriptors of the 0, 50, and 100 score positions. They were then directed to make a pen mark on the line that indicated their response; the distance from zero in mm was the score [26].

### Ocular signs

#### Ocular surface assessment (Efron grading)

Biomicroscopy (Topcon SL-D7) at 16x magnification with a white broad beam at full illumination was used for objective anterior corneal assessment using the 0.1 increment Efron grading scale [27]. The following were assessed: bulbar hyperemia, limbal hyperemia, blepharitis (eyelid redness), meibomian glands, corneal epithelial staining using fluorescein and upper eyelid papillae with lid eversion. A Wratten 12 filter was also utilized for conjunctival epithelial staining and upper eyelid papillae assessment.

#### Tear film break-up time (TBUT)

Tear film quality was assessed using TBUT. Fluorescein was instilled on the subject’s lower palpebral conjunctiva. The participant was instructed to take one slow blink before looking straight ahead without any blinking [28]. The tear film was assessed using a cobalt blue filter with a broad beam at full illumination at 16x magnification under the slit lamp. The time elapsed between the last blink and the first appearance of a disrupted tear film is recorded. This procedure was repeated three times and averaged. TBUT values below 10s were indicative of dry eye.

#### Phenol red test (PRT)

The Phenol Red Thread (PRT) tear test (Zone-Quick, Massachusetts) was used to assess tear quantity. The lower lid was pulled down and the bent edge of the thread was placed within the lower palpebral conjunctival fornix. The participant was asked to look straight ahead and to blink normally for 15 s. The thread was then removed from the subject’s eye and the length of the red portion of the thread from the tip is measured and recorded. Results of ≤5 mm indicate severe dry eye, ≤ 10 mm borderline dry eye and > 10 mm normal [29].

### Statistical analysis

The Shapiro-Wilk test was conducted to determine normality of the data (α =0.05). To compare longitudinal changes between menstrual cycle days, parametric data was analyzed using the paired samples t-test and non-parametric data was analyzed using the Wilcoxon signed rank test. Pearson’s correlation was used to determine associations between variables at each visit. To minimize type II error, Bonferroni corrections were applied. In order to predict ocular health (signs and symptoms of dry eye) based on lifestyle behaviours of healthy young women over the menstrual cycle, we conducted linear regression analysis. Statistical analysis was conducted with SPSS software (SPSS v 25.0; Inc., Armonk, NY). Statistical significance level was set at *p* < 0.05.

## Results

### Participants

Thirty participants were screened. Three participants were ineligible due to abnormal menstrual cycles. One participant withdrew from the study due to personal reasons after attending one visit (at day 7 of the menstrual cycle). A total of 26 participants (mean age = 22.3 ± 3.7 years) with an average menstrual cycle of 28 ± 1 days completed the three visits.

### Changes in ocular sign and symptoms over the menstrual cycle

**Fig. 1:**
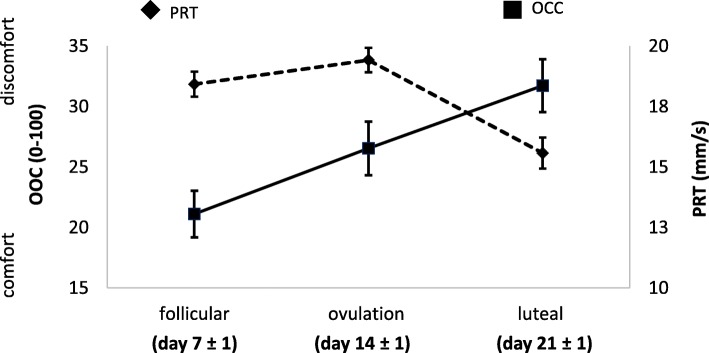
Changes in symptoms (overall ocular comfort score) and tear quantity (phenol read thread values, PRT) across the menstrual cycle. Results are expressed as mean ± SEM

Some signs and symptoms changed over the menstrual cycle and comparisons of days 7, 14 and 21 are shown in Table 1. Blepharitis score was lower at day 7 (0.2 ± 0.2) than day 21 (0.4 ± 0.3) (*p* = 0.003); at day 14, meibomian gland dysfunction (MGD) was lower (0.4 ± 0.4) and PRT scores higher (19 ± 5) compared to day 21 (0.8 ± 0.5, *p* = 0.005; 16 ± 7, *p* = 0.013 respectively); and, OOC scores were higher at day 21 (32 ± 23) than day 7 (21 ± 20) (*p* = 0.030) shown in Fig. 1. Self-reported lifestyle scores did not vary across the menstrual cycle.

### Correlations between lifestyle factors and ocular signs/symptoms over the menstrual cycle

**Fig. 2:**
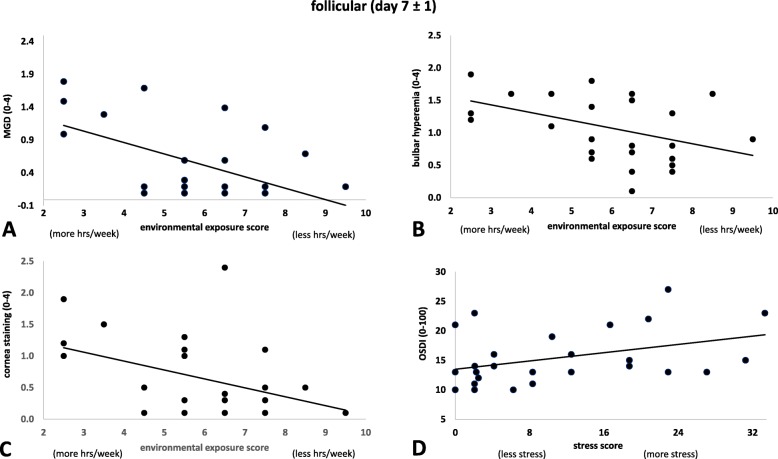
Follicular phase correlations. The association between a lifestyle factor (environmental exposure time) and **a**. meibomian gland dysfunction, **b**. bulbar hyperaemia, and **c**. corneal staining. Lower score indicates more outdoor hrs per week. **d**. The association between a lifestyle factor (stress) and ocular symptoms (OSDI). Lower scores indicate less stress levels per week

The interaction between ocular surface signs/symptoms and lifestyle factors changed over the cycle. At the follicular phase (day 7), lifestyle factors scores related to nutrition and levels of stress were correlated with PRT and OSDI (r = − 0.4, *p* = 0.022; r = 0.4, *p* = 0.045 respectively). Environmental exposure time correlated with bulbar redness (r = − 0.4, *p* = 0.026), MGD (r = − 0.4, *p* = 0.046) and corneal staining (r = − 0.4, *p* = 0.045) (Fig. 2).

**Fig. 3:**
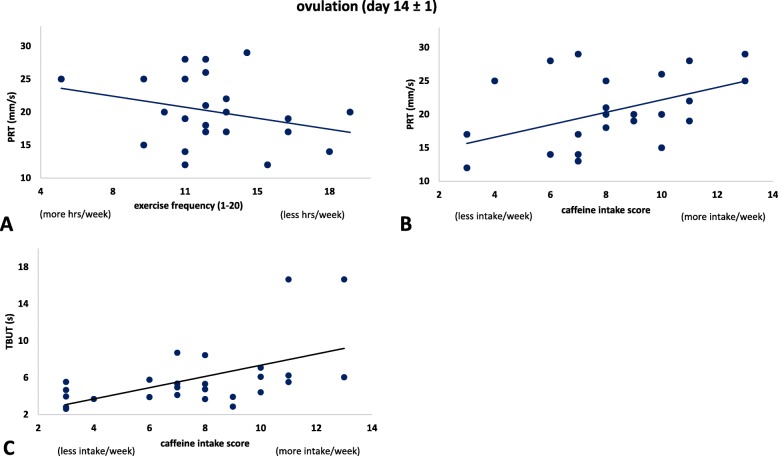
Ovulation phase correlations. **a**. The association between a lifestyle factor (exercise frequency) and ocular sign (tear quantity). Lower score indicates more exercise hrs per week. The association between a lifestyle factor (caffeine intake) and **b**. tear quantity, and **c**. tear quality. Lower score indicates less caffeine intake per week

At the ovulation phase (day 14), the general health score was correlated with OOC scores (r = 0.4, *p* = 0.047). At day 14, exercise frequency correlated with PRT (r = − 0.4, *p* = 0.028) and caffeine intake was also correlated with both; TBUT (r = 0.5, *p* = 0.020) and PRT (r = 0.5, *p* = 0.014) (Fig. 3). At the luteal phase (day 21), there was no correlation between lifestyle factors and dry eye signs or symptoms. The associations between lifestyle factors and ocular sign and symptoms over the menstrual cycle (excluding day 21) are summarised in Table 2.

**Table 2:** Clinical ocular surface characteristics across the menstrual cycle and the association with lifestyle factor scores

Parameter	Scores over menstrual cycle (mean ± SD)			Correlations with lifestyle factors (Pearson’s r)													
	Follicular day 7	Ovulation day 14	Luteal day 21	General health score†		Nutrition		Caffeine intake		Stress levels		Exercise frequency		Cosmetics use		Environment exposure time	
				day 7	day 14	day 7	day 14	day 7	day 14	day 7	day 14	day 7	day 14	day 7	day 14	day 7	day 14
OSDI (0–100)	11 ± 10	11 ± 11	13 ± 11	−0.07	0.05	0.11	−0.12	−0.31	−0.17	0.39^*^	0.26	− 0.26	− 0.14	− 0.02	− 0.02	− 0.07	− 0.12
OOC (0–100)	21 ± 20**	26 ± 23	33 ± 23**	0.05	0.40^*^	−0.05	− 0.25	−0.11	0.05	0.07	−0.10	0.32	−0.21	−0.27	− 0.23	−0.07	0.04
Bulbar Hyperaemia (0–4)	1.1 ± 0.5	1.1 ± 0.5	1.1 ± 0.5	0.25	0.20	−0.03	− 0.03	0.34	0.27	0.07	−0.07	−0.07	− 0.15	0.27	0.30	−0.43^*^	− 0.05
Limbal Hyperemia (0–4)	0.7 ± 0.5	0.7 ± 0.5	0.7 ± 0.4	0.15	0.10	−0.11	− 0.01	0.34	0.09	0.38	0.04	0.06	−0.26	0.06	0.30	−0.36	−0.23
Blepharitis (0–4)	0.2 ± 0.2**	0.2 ± 0.2	0.4 ± 0.3**	0.23	−0.21	− 0.02	−0.13	0.34	−0.04	0.22	−0.13	0.00	0.08	0.25	−0.04	−0.25	− 0.33
MGD (0–4)	0.5 ± 0.6	0.4 ± 0.4*	0.8 ± 0.5*	0.07	−0.19	− 0.00	−0.25	0.12	−0.24	0.24	0.01	0.00	0.11	0.22	0.25	−0.57*	−0.04
Corneal Staining (0–4)	0.6 ± 0.6	0.6 ± 0.6	0.7 ± 0.6	−0.02	0.04	0.05	−0.22	−0.04	− 0.01	−0.09	− 0.12	−0.04	0.21	−0.03	0.34	−0.40*	0.05
Papillae (0–4)	1.1 ± 0.9	0.9 ± 0.7	1.0 ± 0.6	0.00	0.15	−0.14	− 0.19	−0.00	− 0.10	−0.24	0.19	0.07	0.17	−0.44*	0.21	−0.28	0.00
TBUT (s)	5.8 ± 2.9	5.4 ± 2.8	4.8 ± 2.3	−0.21	− 0.23	−0.34	− 0.02	0.11	0.45^*^	−0.30	− 0.08	−0.10	− 0.20	−0.04	− 0.23	−0.18	− 0.08
PRT (mm/s)	18 ± 5	19 ± 5*	16 ± 7*	−0.16	− 0.04	−0.44^*^	− 0.05	0.32	0.48^*^	0.01	0.35	−0.15	−0.43^*^	0.20	−0.02	− 0.04	−0.18

Results of the ocular surface assessment are expressed as mean ± SD. A two-tailed ⍺ = 0.05 level of significance was considered for all analyses. *Significant difference of day 14 vs 21; **day 7 vs 21. Lower values of lifestyle factor indicated healthier choices. Pearson correlation was used to determine the correlation between lifestyle factors and sign/symptoms of dry eye. Significant correlations are starred *p < 0.05.†general health scores were obtained only at baseline (day 14 represents the values of parameters taken at day 14)

*OSDI* ocular surface disease index, *OOC* overall ocular comfort, *TBUT* tear break-up time, *PRT* phenol red thread, *MGD* meibomian gland dysfunction

Significance changes and associations are shown in bold

### Lifestyle predictors for sign and symptoms of dry eye over the menstrual cycle

Linear regression analysis was calculated to predict ocular health (signs and symptoms) based on lifestyle behaviours of young healthy women. All associations shown in Table 3 were significant. The strongest regression equation was F (1,25) = 11.179, *p* = 0.003, with an R of 0.556, indicating that participant’s predicted MGD at day 7 of the menstrual cycle is equal to 1.479 + (− 0.173 outdoor exposure score) when MGD is measured using Efron grading scale. Participant’s MGD increased 0.15 for each hour of reported outdoors hours.

**Table 3:** Linear regression analysis with ocular health (signs and symptoms) as predictors based on lifestyle behaviours

Predictor	Factor	Significant regression equation		R
		Day 7	Day 14	
General health score	OCC (0–100)		F (1,24) = 4.385, *p* = 0.047	0.393
Nutrition	PRT (mm/s)	F (1,25) = 6.008, *p* = 0.022		0.440
Caffeine intake	TBUT (s)		F (1,24) = 6.242, *p* = 0.020	0.454
	PRT (mm/s)		F (1,24) = 6.987, *p* = 0.014	0.475
Stress levels	OSDI (0–100)	F (1,25) = 4.461, *p* = 0.045		0.389
Exercise frequency	PRT (mm/s)		F (1,24) = 5.495, *p* = 0.028	0.432
Cosmetic use	Papillae (0–4)	F (1,25) = 5.991, *p* = 0.022		0.440
Environmental exposure time	Bulbar hyperaemia (0–4)	F (1,25) = 5.600, *p* = 0.026		0.428
	MGD (0–4)	F (1,25) = 11.179, *p* = 0.003		0.556
	Corneal staining (0–4)	F (1,25) = 4.797, *p* = 0.038		0.401

*OSDI* ocular surface disease index, *OOC* overall ocular comfort, *TBUT* tear break-up time, *PRT* phenol red thread, *MGD* meibomian gland dysfunction

## Discussion

This observational study explored, for the first time, the effect of modifiable lifestyle factors of dry eye over the menstrual cycle in a group of young healthy adults. This result showed that the ocular surface has greater sensitivity to modifiable risk factors at day 7 and 14 compared to day 21; there were more associations between healthy lifestyle choices and ocular signs and symptoms during the follicular phase (day 7) than the ovulation and luteal phases of the menstrual cycle. The overall ocular comfort measured using the visual analog scale (0–100) indicated greater comfort at day 7.

However, ocular signs such as tear volume, blepharitis and MGD scores were worse at day 7 compared to day 21, the opposite trend to the that for comfort scores over the cycle. The disagreement between signs and symptoms of dry eye measured at one-time point is in line with the evidence from previous studies [30, 31]. During the follicular phase of the menstrual cycle, both concentration levels of progesterone and estrogen are relatively low compared to ovulation and the luteal phases [32] and these concentration levels may play a role in ocular symptoms and signs. Animal models of Sjögren’s syndrome have shown that the absence of oestrogenic influence in lacrimal glands leads to regressive, inflammatory changes in the tissue, while oestrogen administration prevents or reverses these changes and promotes lacrimal secretion [33, 34]. In support of this finding, associations between low oestradiol, oestrone and testosterone levels and poor tear osmolarity in postmenopausal women with severe evaporative dry eye have previously been found [35].

Another possible factor to explain the increase in ocular discomfort scores at day 21 is the manifestation of premenstrual syndrome, which is characterized by cyclical changes in psychological and physical symptoms related to the formation of the corpus luteum and the fluctuations of the major steroid hormones including estradiol and progesterone [36]. A higher sensitivity to pain stimuli is observed during the luteal phase of the menstrual cycle, which probably results from a reduction in the descending inhibitory control on spinal nociceptive flexion reflex [37]. This explain the disconnect between ocular signs and symptoms of dry eye at day 7.

Premenstrual syndrome has an increased effect in young females especially in those under increased stress and experiencing lack of sleep [38]. The study participants of this investigation were young university students and although the stress levels of these students were relatively low as indicated by the scores of the questionnaire, there was a significant correlation between stress levels and ocular symptoms measure with OSDI indicating that the higher stress levels were associated with grater ocular discomfort (Fig. 2).

At the follicular phase, ocular signs such as redness, meibomian gland dysfunction and corneal staining correlated with environmental exposure time indicating that more time indoors was indicative of a healthier ocular surface. Several environmental factors have been suggested to impact dry eye diseases, such as air pollution, wind, low humidity and high altitude [3]. This study was conducted in the urban region of Brisbane city. Overall, the quality of Brisbane air meets national standards, but occasionally higher levels of smog and particle pollution are experienced. Smog events occur from the interactions between air pollutants in hot, low-wind conditions [39]. These factors can contribute to dry eye and may, in part, explain the results in this study, however, the reason that these correlations were only observed at the follicular phase is unknown.

Meibomian gland dysfunction evaluation using the grading Efron scale showed to be the strongest ocular sign predictor based on the environmental exposure time during the follicular phase of the menstrual cycle in young healthy women. These results are consistence with the physiopathology of MGD as temperature, humidity, and air quality are known risk factors for the disease [40]. However, the underlying reason for this correlation being present only during the follicular phase is unknown.

At the ovulation phase, high levels of estrogen in combination with caffeine intake demonstrate an association pattern indicating that the more caffeine intake the better tear quantity and quality. Caffeine is a psychoactive substance that has been claimed to have effects on some tear film dynamics [3, 41, 42]. Like other methylxanthines, caffeine’s inhibition of 3,5-cyclic nucleotide phosphodiesterase (cAMP-PDE) can explain its stimulatory effect on the tear gland [43]. It appears that the higher levels of estrogen potentiate the effect of caffeine, by what mechanism this occurs requires investigation.

Increased exercise frequency was also associated with improved tear quantity during the ovulation phase. The Osaka study, which comprised a cross-sectional investigation of dry eye among office workers, found several new systemic health factors associated with dry eye disease, such as metabolic syndrome, low exercise habit, sedentary lifestyle and poor sleep quality. The results showed that a high level of physical activity was associated with a low risk of dry eye and that sedentary behavior was a risk factor [44]. Animal work also suggests that excess oxidative stress is systemically associated with lacrimal dysfunction [45] and calorie restriction maintains tear secretion and reduces oxidative stress [46].

At the luteal phase, there was no correlation between modifiable lifestyle risk factors and sign and symptoms of dry eye. There is a lack of evidence in the literature with regards the effects of progesterone levels and the ocular surface. However, Golebiowski and co-workers determined no correlation between plasma progesterone levels and ocular surface sign and symptoms in both male and females, but suggested that lower levels of progesterone affect the ocular surface with age [36].

In animal work using female rats, levels of LH increase at the beginning of the luteal phase. This hormone induces the desensitisation of brain opioid receptors, resulting in increased pain sensitivity [47, 48]. Reascent human studies in dry eye suggest that it is likely that a subset of dry eye patients have neuropathic pain and central sensitization [49]. These patients are likely to be more resistant to topical therapy directed at optimizing the ocular surface. The increase of this receptors may explain increase in ocular discomfort during day 21 and therefore, the lack of association between with ocular signs and symptoms during the luteal phase.

The significant variation in ocular symptomatology from day 7 to day 21 demonstrated increased levels of discomfort by approximately 12%, these values suggest that females who reported moderate symptoms of dry eye levels during day 7 and 14 changed to report severe levels on day 21. This physiological changes in symptoms may impact diagnosis of dry eye indicating that young females are likely to report severe dry eye symptoms during the luteal phase of the menstrual cycle. Therefore, this study suggest that menstrual cycle should be taken in account for future assessment of dry eye symptomatology in young healthy females.

The effect of variation in symptomatology was observe in 71% of the participants indicating that the effect of luteal phase of the menstrual cycle clearly plays a role in ocular comfort scores. The reason why 29% of the participants show no variation or opposite effect is not clearly understood and future research should be done in this area.

The influence of hormonal changes over the menstrual cycle on other sensory systems such as audiological or olfactory have previously been reported. Increased progesterone in luteal phase can lead to abnormal hearing whereas improvement auditory performance is seen in the follicular phase [50]. Olfactory sensitivity threshold during the menstrual cycle is perceived faster around ovulation and slower during the follicular phase possibly due to the changes of cortisol levels over the period [51].

Differentiating between the female population within dry eye and evaluating for the presence of potential effect of ocular symptoms related to menstrual cycle will be critical to individualizing the treatment and diagnosis of dry eye in young females.

Providing new evidence of young healthy individuals, during the menstrual cycle, to increase knowledge and understanding of ocular health comparable to the existing evidence of individuals under the spectrum of ocular surface diseases is the main strength of this study. However, larger cohorts and more reliable measurement of hormone levels are recommended for future studies.

## Conclusions

The effect of lifestyle factors appeared to be more pronounced during the ovulation phase compared to the follicular and luteal phases of the menstrual cycle in young healthy women. Misalignment of these factors with the ocular health during the luteal phase could be attributed to the central sensitization and elevated levels of progesterone. Natural hormonal changes occurring during the menstrual cycle should be considered when assessing the ocular surface in young healthy women.

## Data Availability

The datasets used and/or analysed during the current study are available from the corresponding author on reasonable request.

## Abbreviations

cAMP-PDE: caffeine’s inhibition of 3,5-cyclic nucleotide phosphodiesterase
DEWSII: Dry eye workshop
FSH: Follicle stimulating hormone
LH: Luteinising hormone
MGD: Meibomian gland dysfunction
OOC: Overall ocular comfort
OSDI: Ocular surface disease index
PRT: Phenol red thread
TBUT: Tear break-up time
TFOS: Tear Film and Ocular Surface Society
